# Maintenance of *Trypanosoma cruzi*, *T. evansi* and *Leishmania* spp. by domestic dogs and wild mammals in a rural settlement in Brazil-Bolivian border

**DOI:** 10.1016/j.ijppaw.2018.10.004

**Published:** 2018-10-17

**Authors:** Grasiela Edith de Oliveira Porfirio, Filipe Martins Santos, Gabriel Carvalho de Macedo, Wanessa Teixeira Gomes Barreto, João Bosco Vilela Campos, Alyssa C. Meyers, Marcos Rogério André, Lívia Perles, Carina Elisei de Oliveira, Samanta Cristina das Chagas Xavier, Gisele Braziliano de Andrade, Ana Maria Jansen, Heitor Miraglia Herrera

**Affiliations:** a Programa de Pós-Graduação em Ciências Ambientais e Sustentabilidade Agropecuária, Universidade Católica Dom Bosco, Tamandaré Avenue, 6000. Jardim Seminário, Cep 79117-900, Campo Grande, Mato Grosso do Sul, Brazil; b Programa de Pós-Graduação em Ecologia e Conservação, Universidade Federal de Mato Grosso do Sul, Costa e Silva Avenue, Cep 79070-900, Campo Grande, Mato Grosso do Sul, Brazil; c Department of Veterinary Integrative Biosciences, Texas A&M University, 402 Raymond Stotzer Parkway, 4458, College Station, Texas, USA; d Universidade Estadual Paulista (Unesp), Faculdade de Ciências Agrárias e Veterinárias, Prof. Paulo Donato Castelane Street, Cep 14884-900, Jaboticabal, São Paulo, Brazil; e Laboratório de Biologia de Tripanosomatídeos, Instituto Oswaldo Cruz, Fundação Oswaldo Cruz, Brazil Avenue, 4365, Manguinhos, Rio de Janeiro, Rio de Janeiro, Brazil

**Keywords:** Canine, Neglected diseases, Pantanal, Sentinels hosts, Trypanosomatids

## Abstract

Domestic dogs are considered reservoirs hosts for several vector-borne parasites. This study aimed to evaluate the role of domestic dogs as hosts for *Trypanosoma cruzi*, *Trypanosoma evansi* and *Leishmania* spp. in single and co-infections in the Urucum settlement, near the Brazil-Bolivian border. Additionally, we evaluated the involvement of wild mammals’ in the maintenance of these parasites in the study area. Blood samples of dogs (n = 62) and six species of wild mammals (n = 36) were collected in July and August of 2015. The infections were assessed using parasitological, serological and molecular tests. Clinical examination of dogs was performed and their feeding habits were noted. Overall, 87% (54/62) of sampled dogs were positive for at least one trypanosomatid species, in single (n = 9) and co-infections (n = 45). We found that 76% of dogs were positive for *T. cruzi*, four of them displayed high parasitemias demonstrated by hemoculture, including one strain types TcI, two TcIII and one TcIII/TcV. Around 73% (45/62) of dogs were positive to *T. evansi*, three with high parasitemias as seen by positive microhematocrit centrifuge technique. Of dogs sampled, 50% (31/62) were positive for *Leishmania* spp. by PCR or serology. We found a positive influence of (i) *T. evansi* on mucous pallor, (ii) co-infection by *T. cruzi* and *Leishmania* with onychogryphosis, and (iii) all parasites to skin lesions of sampled dogs. Finally, feeding on wild mammals had a positive influence in the *Leishmania* spp. infection in dogs. We found that 28% (5/18) coati *Nasua nasua* was co-infected for all three trypanosamatids, demonstrating that it might play a key role in maintenance of these parasites. Our results showed the importance of Urucum region as a hotspot for *T. cruzi*, *T. evansi* and *Leishmania* spp. and demonstrated that dogs can be considered as incidental hosts.

## Highlights

• Observation of high occurrence of dogs co-infected by trypanosomatids.
• Dogs infected by TcI, TcIII and TcIII/TcV.
• *Nasua nasua* is a key species in the sylvatic cycles of trypanosomatids.
• Direct effect of trypanosomatids' infection in clinical signs of dogs.
• Dogs as sentinels to human infection in the Brazil-Bolivian border.

## Graphical abstract

**Figure:**
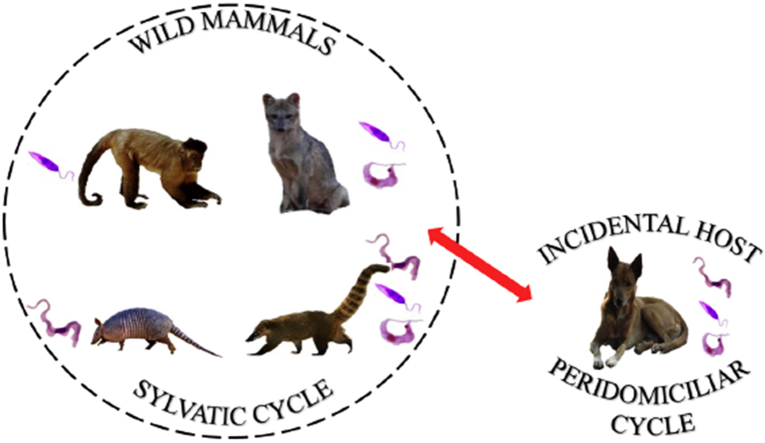

## 1 Introduction

In cross-border regions between underdeveloped countries, the control of diseases caused by multi-host parasites is impaired due to differences in social, cultural, economic, environmental and sanitary regulations. In many cases of dry borders, the free traffic of people and domestic animals, including dogs, intensify the transboundary sanitary problems, and therefore increase the risk of emergence of zoonotic diseases (Daszak et al., 2000; Weinberg et al., 2003; Esteve-Gassent et al., 2014).

Dogs can act as sentinels for some parasitic infections that occur in the sylvatic environment (Castañera et al., 1998; Roque and Jansen, 2008; Rabinowitz et al., 2009; Wang et al., 2012). Predation and agonistic encounters between dogs and wild mammals have been reported as a source of spill-over and spill back for parasitic infection in rural areas (Daszak et al., 2000; Thompson et al., 2009). In rural settlements, dogs are used to guard the properties or for companionship in the field. Dogs are also frequently used for hunting, where exposure to pathogens could result from contact or consumption of wild animals.

Dogs are important domestic reservoirs for *Trypanosoma cruzi* and *Leishmania* spp., constituting a matter of public health concern worldwide (Gürtler et al., 1993; Maia-Elkhoury et al., 2008; Esch and Petersen, 2013; Gürtler and Cardinal, 2015). The Brazil state of Mato Grosso do Sul is enzootic for *T. cruzi*, *Leishmania* spp. and *Trypanosoma evansi* which are the etiological agents of Chagas disease, leishmaniasis and animal trypanosomiasis, respectively (Mello et al., 1988; Silva et al., 1995; Herrera et al., 2011; Santos et al., 2015). Furthermore, dogs have been suggested to provide a link between domestic and wild *T. cruzi* transmission cycles (Roque and Jansen, 2008), and have useful as sentinels for *T. cruzi* in enzootic areas (Roque and Jansen, 2008; Ramírez et al., 2013; Xavier et al., 2014). Nomenclature of *T. cruzi* is complex, and currently seven discrete typing units (DTUs) are recognized TcI-TcVI and TcBat (Zingales et al., 2012; Barros et al., 2017). Dogs are considered the main domestic reservoir for leishmaniasis, due to their close contact with humans and presence of high numbers of parasites in the skin (Reis et al., 2010; Nunes et al., 2016; Sevá et al., 2016).

Trypanosomiasis caused by *T. evansi* has been reported in many species of wild mammals and domestic mammals at the Pantanal region (Nunes et al., 1993; Herrera et al., 2004). Like horses, dogs are particularly susceptible to infection by *T. evansi* (Hoare, 1972; Franke et al., 1994; Herrera et al., 2004). Trypanosomiasis is characterized mainly by acute, progressive and severe anemia in dogs and horses (Silva et al., 1995; Aquino et al., 2002).

These three species of trypanosomatids are multi-host parasites, capable of parasitizing a large number of domestic and wild mammalian species (Herrera et al., 2011; Jansen et al., 2015). Although *T. cruzi*, *T. evansi* and *Leishmania* spp. have been reported in the Mato Grosso do Sul state, their prevalence and maintenance hosts in the rural settlements of Corumbá city is unknown. This study aimed to evaluate the role of domestic dogs and wild mammals as hosts for *T. cruzi*, *T. evansi* and *Leishmania* spp., in the maintenance of these parasites in the Urucum rural settlement, near the Brazil-Bolivian border. Additionally we aimed to evaluate the influence of single or co-infections on clinical aspects of dogs.

## 2 Material and methods

### 2.1 Study area

**Fig. 1:**
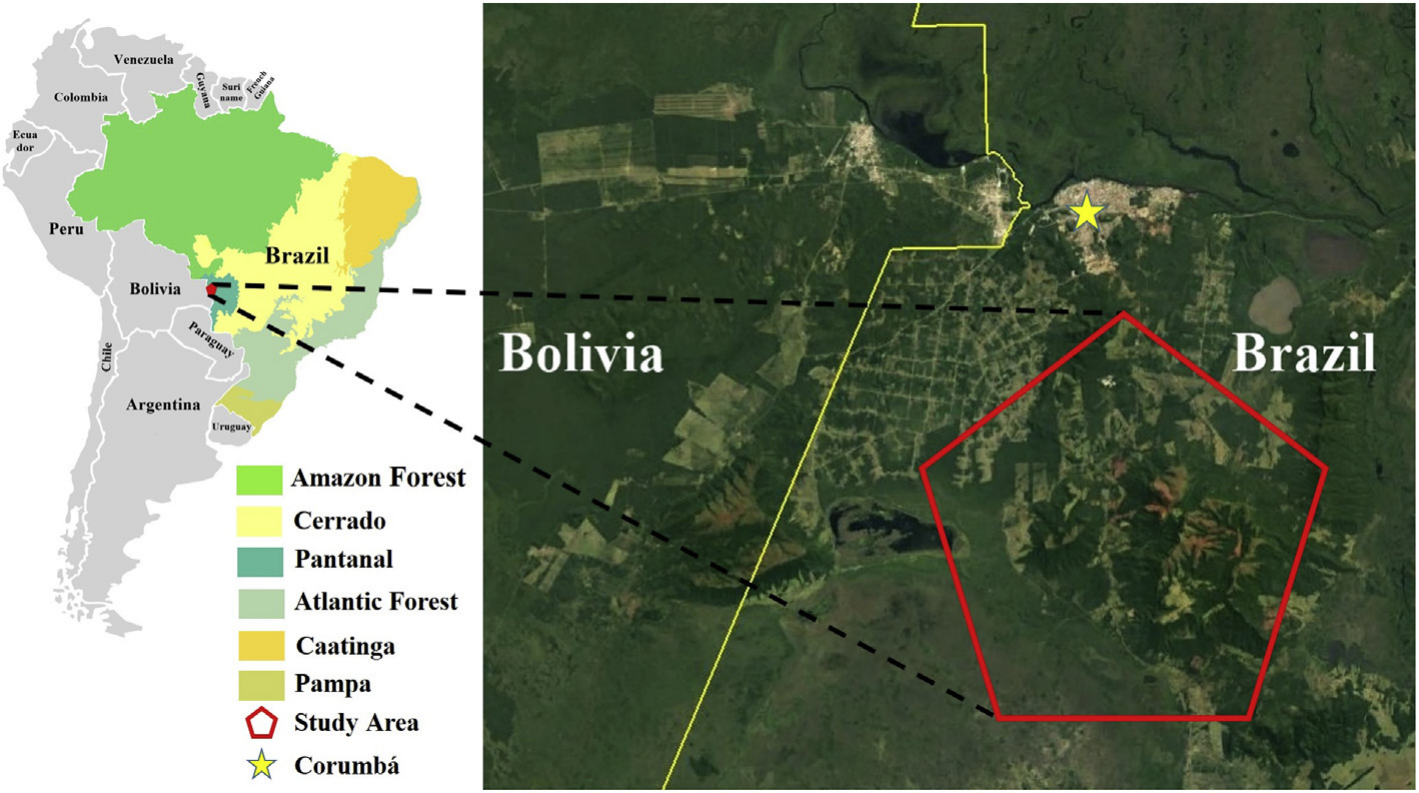
The Brazil-Bolivian border and Urucum settlement (Corumbá, MS) demonstrating the site of collections.

The study was carried out in the Urucum rural settlement, located near to the Brazil-Bolivian border, approximately 18 km from the urban area of Corumbá, Mato Grosso do Sul state (Fig. 1). The total area is 1979 hectares, which is divided in 84 small properties. Approximately 87 families live in Urucum rural settlement. The main economic activities are cassava and vegetables growing, dairy farming and breeding of chickens, pigs and cattle. A census survey carried out by the Zoonoses Control Center (ZCC) estimated domestic dogs’ population in approximately 300 animals (ZCC, Unpublished results).

### 2.2 Blood sample of dogs

Sampling was conducted in July and August of 2015. Approximately 4 ml of venous blood was collected from each animal using sterile techniques: 2 ml were stored in a tube with Ethylenediamine Tetra acetic Acid (EDTA) for parasitological and molecular testing, and the other 2 ml were stored in tubes without EDTA for serological testing.

### 2.3 Capture and blood sampling of wild mammals

From July to August 2015 thirty tomahawk traps were deployed (90 × 45 x 50 Equipos Fauna^®^) in the region. Traps were placed on both the ground and in trees of forested areas. Traps set on the ground were baited with bacon, while traps set on the trees were baited with bananas. Traps were checked daily during ten days at two surveys, totaling a sample effort of 300 Trap-night. Animals were immobilization using a combination of Tiletamine and Zolazepam (Zoletil 50^®^), with doses varying according to the species and weight of captured animal. Using sterile techniques approximately 4 ml of venous blood was collected from each animal, which were divided into EDTA and serum tubes.

### 2.4 Diagnostic of trypanosomatids

Infections by *T. cruzi*, *T. evansi* and *Leishmania* spp. were assessed in domestic dogs and wild mammals using parasitological, molecular and serological tests. Parasitological testing for *T. cruzi* and *Leishmania* spp. was carried out using hemoculture (HC) and inoculating 300 μl of blood in Novy McNeal Nicole (NNN) medium with Liver Infusion Tryptose (LIT) overlay and NNN with Schneider biphasic medium, in duplicate. Hemoculture tubes were incubated at 27 °C during 30 days and monitored for parasite development once a week. When epimastigotes were present, they were subjected to DNA extraction using the previously described phenol-chloroform method (Vallejo et al., 1999), and deposited at the “Coleção de *Trypanosoma* de Mamíferos Silvestres, Domésticos e Vetores, Fiocruz – COLTRYP” (Oswaldo Cruz Foundation, Rio de Janeiro - RJ/Brazil). To determine the DTU of *T. cruzi* samples, all NNN + Lit positives were subjected to multiplex PCR amplification of the non-transcribed spacer of the mini-exon gene (SL-IR) according to Fernandes et al. (2001) and Westenberger et al. (2005). Each reaction included negative and positive control samples from *T. cruzi* strains representing the six DTUs. For *T. evansi* we used Microhematocrit Centrifuge Technique (MHCT) to determine if an animal was parasitemic (Woo, 1970).

For molecular testing genomic DNA was extracted from 200 μl of total blood using the QIAamp Blood DNA Mini Kit (Qiagen, Hilden, Germany) according to the manufacturer's instructions. Detection of *Trypanosoma* sp. infection in host blood was performed by nested Polymerase Chain Reaction (nPCR) that targeted a variable region of the trypanosome 18S rRNA gene, with external primers TRY927F and TRY927R, and internal primers SSU561Fand SSU561R, according to Smith et al. (2008). Positive samples were further subjected to D71 and D72 primers which amplify a conserved sequence of the large subunit of the ribosomal DNA gene (24Sα rDNA), to test for *T. cruzi* (Souto and Zingales, 1993). To test for *T. evansi,* positive samples were also run on TBR1 and TBR2 primers which amplify sequence of mini-chromosome satellite DNA for *T. evansi* (Masiga et al., 1992). Finally, samples were run using *Leishmania* specific primers A and B targeting kDNA of *Leishmania* sp. (Schubach et al., 1998). Each reaction included negative and positive control samples from *Trypanosoma* sp., *T. cruzi*, *T. evansi* and *Leishmania* spp. PCR products were visualized in 2% agarose gel after ethidium bromide staining under ultraviolet light.

Indirect Fluorescent Antibody Test (IFAT) and the Enzyme-Linked Immunoabsorbent Assay (ELISA, Biomanguinhos, Rio de Janeiro-RJ, Brazil) were used for detection of anti-*T. cruzi* and anti-*Leishmania* IgG antibodies as previously described by Xavier et al. (2014) for domestic dogs, crab-eating-fox (*Cerdocyon thous*), and coatis (*Nasua nasua*). Serological tests for *T. cruzi* and *Leishmania* spp. were not carried out for primates (*Sapajus cay*), agoutis (*Dasyprocta azarae*) and armadillos (*Euphractus sexcinctus* and *Dasypus novemcinctus*) because conjugated fluoresceine antibodies against these species IgG have not yet been developed.

For the detection of anti-*T. evansi* IgG antibodies we used an IFAT assay on dogs and *C. thous* only (Aquino et al., 2010). Currently there are no conjugated fluoresceine antibodies against IgG for *N. nasua*, *S. cay*, *D. azarea*, *E. sexcinctus* and *D. novemcinctus*. The cut-off value for the IFAT was 1∶40. The cut-off value for the ELISA was defined as the mean optical absorbance of the negative controls +20%. To each reaction plate, 2 positive and 2 negative control sera were run.

In the present study we considered high parasitemias (patent infection) the positivity in the parasitological tests HC and MHCT. An animal was considered infected when a positive result was found on any of the diagnostic tests used: parasitological, serology and molecular.

### 2.5 Physical examination of dogs

Clinical signs suggestive of trypanosomiasis (weight loss, lymphadenomegaly, onychogryphosis, mucous pallor, skin lesions and ocular signs) were evaluated by a veterinarian and noted for each dog. In addition, owners were asked about feeding habits and contact with wild mammals.

### 2.6 Statistical analysis

Single and co-infections were expressed as frequency of occurrence (percentage of single and co-infection regarding all animals sampled). Due to the number of samples collected, statistical analysis was performed only on dogs.

Path Analysis was used to estimate the influence in the relationship between positive dogs and clinical signs, contact with wildlife and feeding on wildlife. The variables were considered to be statistically significant when values of *p* were ≤0.05.

To test possible associations among the three trypanosomatids evaluated, we used a Chi-squared test and statistical significance was measures at *p* ≤ 0.05. Data were analyzed using R 3.4.2 (R Development Core Team, 2015).

## 3 Results

### 3.1 Dogs

**Fig. 2:**
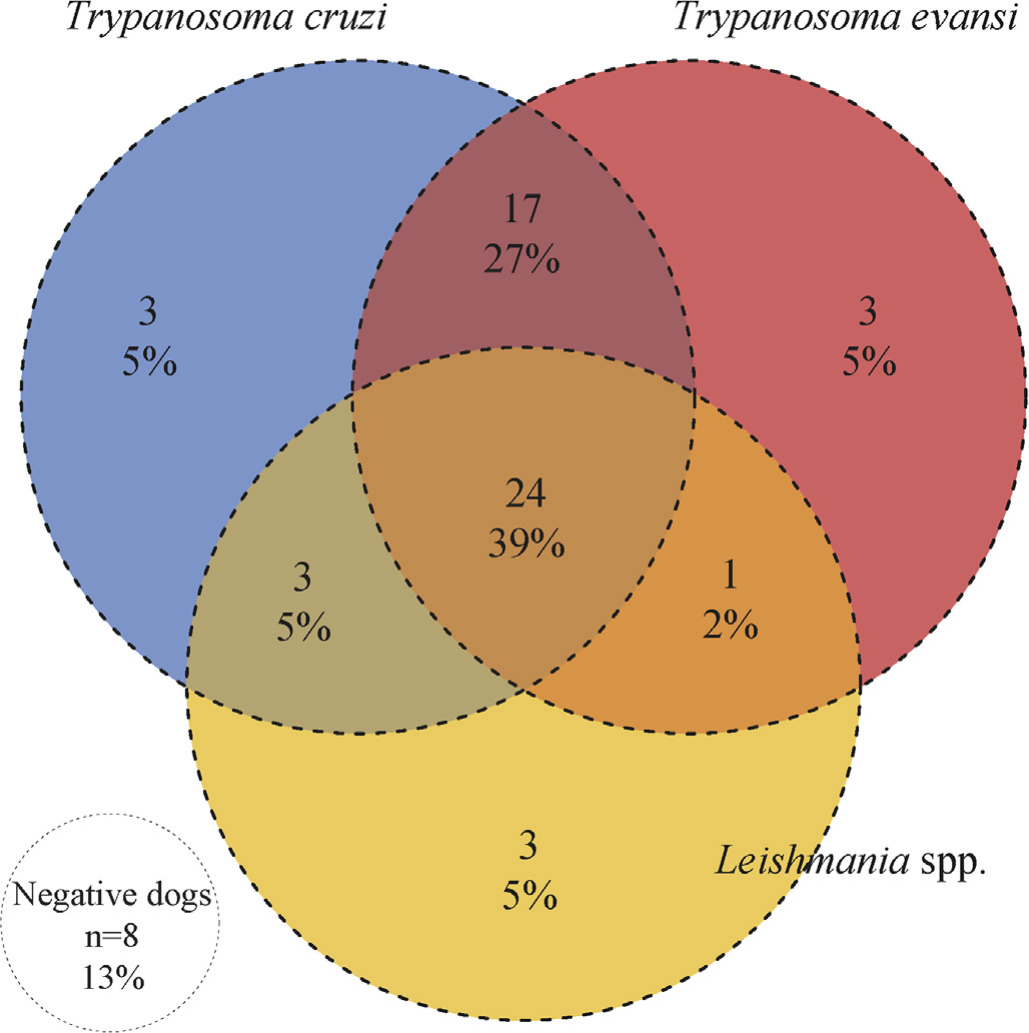
Three-way Venn diagram illustrating coinfection, single infection or no infection of *T. cruzi, T. evansi,* and *Leishmania* spp. in 62 dogs from the Urucum settlement along the Brazil-Bolivia border. Total numbers and percentages are presented.

Overall, 87% (54/62) of sampled dogs were positive for at least one of trypanosomatids species, in single (n = 09) and co-infections (n = 45) (Fig. 2). Forty seven dogs (76%) were positive for *T. cruzi*. We found high parasitemias in four (6%) examined dogs, which were also positive on both PCR and serological tests. Genotyping from these dogs revealed one TcI, two TcIII, and one co-infection of TcIII/TcV. By PCR or serological tests, 50% (31/62) and 53% (33/62) of dogs’ were *T. cruzi* positive, respectively (Table 1). Discordant results were found, where 19% (12/62) of dogs tested were negative by serology and positive by PCR, while 23% (14/62) were positive by serology and negative by PCR (Table 2).

**Table 1:** Number of positive samples for hemoculture, Microhematocrit Centrifuge Technique (MHCT) molecular and/or serological tests performed for *Trypanosoma cruzi*, *Trypanosoma evansi* and *Leishmania* spp. in samples of 62 dogs surveyed in the Urucum settlement, Corumbá, Mato Grosso do Sul, Brazil in 2015.

Parasite	Hemoculture No.%	MHCT No.%	Molecular Test No.%	Serological Test No.%
*Trypanosoma cruzi*	4 (6)		31 (50)	33 (53)
*Trypanosoma evansi*		3(5)	43 (69)	18 (29)
*Leishmania* spp.			22 (35)	16 (26)

**Table 2:** Patterns of infection evidenced by serological and PCR tests carried out to detect *Trypanosoma cruzi*, *Trypanosoma evansi* and *Leishmania* spp. in blood samples of dogs and *Nasua nasua* surveyed in the Urucum settlement, Corumbá, Mato Grosso do Sul, Brazil.

Hosts	Serology- PCR-No.%	Serology- PCR + No.%	Serology + PCR-No.%	Serology + PCR + No.%
**Dogs**				
*Trypanosoma cruzi*	17 (27)	12 (19)	14 (23)	19 (31)
*Trypanosoma evansi*	15 (24)	29 (47)	4 (6)	14 (23)
*Leishmania* spp.	31 (50)	15 (24)	9 (15)	7 (11)
***Nasua nasua***				
*Trypanosoma cruzi*	11 (61)	0	4 (22)	3 (17)
*Leishmania* spp.	9 (50)	1 (05)	5 (28)	3 (17)

Infection with *T. evansi* was found in 45 dogs (73%). We found three dogs with high parasitemias using the MHCT test (Table 1). All dogs with high parasitemias for *T. evansi* were also positive by PCR and serology. By serology alone 69% (43/62) of dogs were positive and by PCR 29% (18/62) of dogs’ were positive (Table 1). Results for *T. evansi* testing also demonstrated discordance; 47% (29/62) of dogs were serology negative and PCR positive, while 6% (4/62) tested positive by serology and negative by PCR (Table 2).

For *Leishmania* spp. we observed 31 dogs (50%) positive. All examined dogs had negative culture results (Table 1). The PCR and serological testing for *Leishmania* spp. demonstrated that 35% (22/62) and 26% (16/62) of dogs’ were positive, respectively (Table 1). Additionally, we observed 24% (15/62) of dogs were serology negative and PCR positive, while 15% (9/62) were serology positive and PCR negative for *Leishmania* spp. (Table 2).

We found 39% (24/62) of dogs were co-infected with all three trypanosomatids, 34% (21/62) of dogs were co-infect with two and 15% (9/62) positive for one parasite. In addition, 13% (8/62) of dogs were negative for all parasites (Fig. 2). Furthermore, we observed no significant association between co-infection (χ^2^ = 5.0509; df = 4; *p* = 0.2821).

**Fig. 3:**
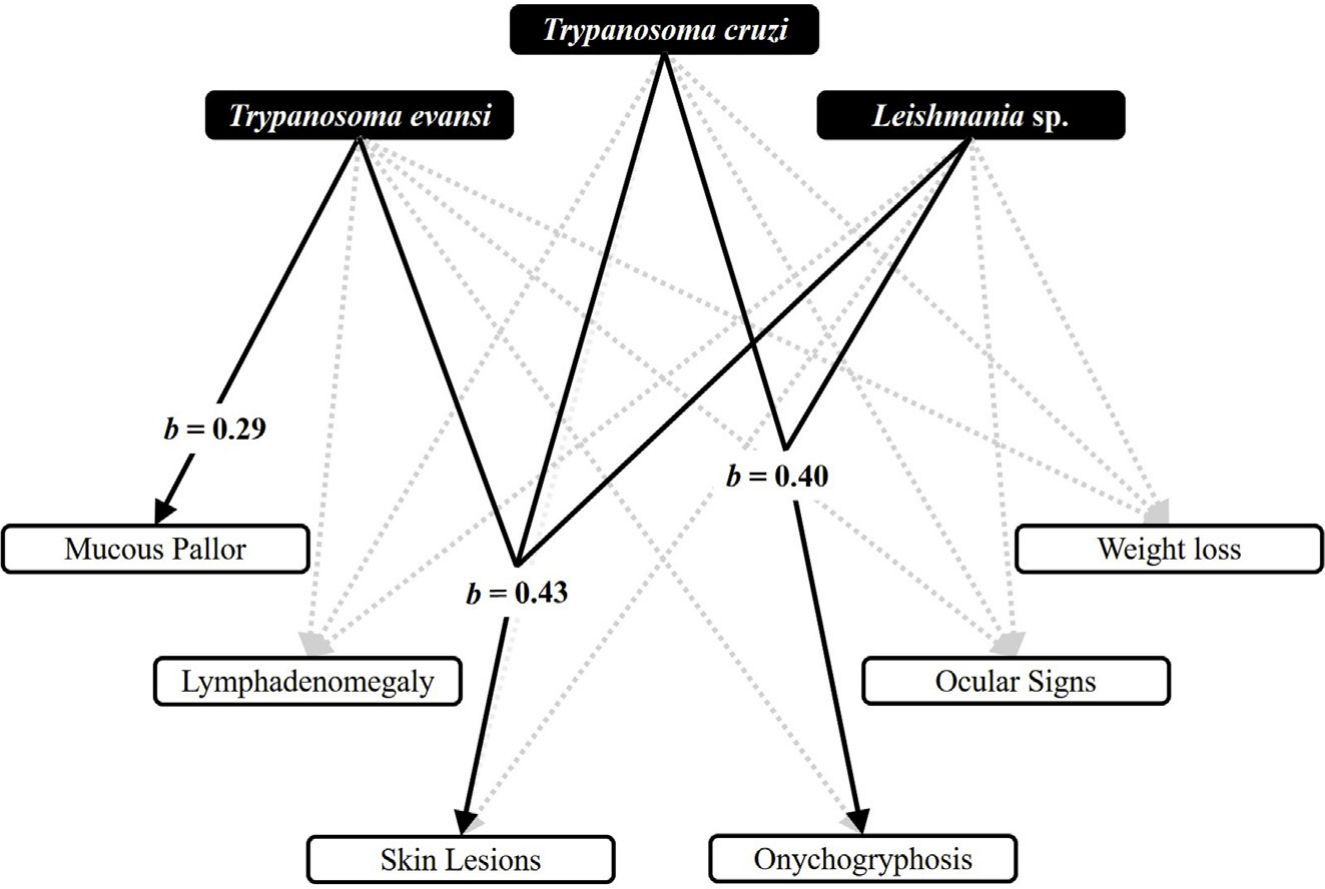
Path analysis on the influences of infections in relation to physical examination of dogs surveyed at Urucum settlement, Corumbá, Mato Grosso do Sul, Brazil in 2015.

**Fig. 4:**
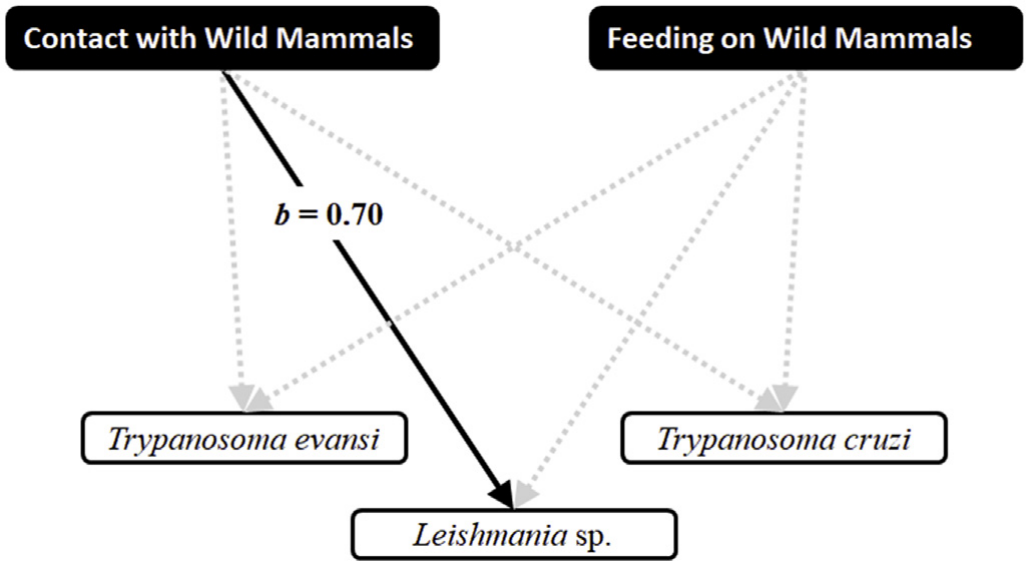
Path analysis on the influences of contact and feeding on wild mammals in relation to infections of dogs surveyed at Urucum settlement, Corumbá, Mato Grosso do Sul, Brazil in 2015.

Among 62 dogs examined, we observed that 35.5% (22/62) showed weight loss, 32.3% (20/62) mucous pallor, 16.1% (10/62) ocular lesions, 33.9% (21/62) skin lesions, 50% (31/62) lymphadenopathy, and 3.2% (2/62) onycogriphosis. The Path Analysis supported that (a) *T. evansi* had a positive influence on the proportion of dogs with mucous pallor (path coefficient of 0.29, p < 0.05); (b) co-infection by *Leishmania* spp. and *T. cruzi* had a positive influence on the proportion of dogs with onychogryphosis (path coefficient of 0.40, p < 0.05); and (c) co-infection by *Leishmania* spp., *T. cruzi* and *T. evansi* had a positive influence on the proportion of dogs with skin lesions (path coefficient of 0.43, p < 0.05) (Fig. 3). Moreover, half of dogs (31/62) which had contact with wild mammals and 40.3% (25/62) were seen feeding on wild mammals as agoutis, *N. nasua*, *S. cay*, *E. sexcinctus* and *D. novemcinctus*. Finally, the Path Analysis demonstrated that the behavior of feeding on wild mammals had a positive influence on infection with *Leishmania* spp. (path coefficient of 0.70, p < 0.05) (Fig. 4).

### 3.2 Wild mammals

A total of 36 wild mammals from six species and four orders were trapped: *N. nasua* (n = 18), *S. cay* (n = 9), *C. thous* (n = 5), *E. sexcinctus* (n = 2), *D. novemcinctus* and *D. azarae* (n = 1).

Seven *N. nasua* (39%) and two *C. thous* (40%) were positive for *T. cruzi*. All wild mammals examined were negative by hemoculture. By *T. cruzi* PCR only 17% (3/18) of *N. nasua* were positive, while all other wild mammals were negative. The serological tests for *T. cruzi* showed that 39% (7/18) of *N. nasua* and 40% (2/5) of *C. thous* tested positives. All *N. nasua* that were PCR positive were also serology positive. Additionally, we found that 22% (4/18) of *N. nasua* examined were positive by serology and negative by PCR (Table 2).

Infection with *T. evansi* was found in 50% *N. nasua* (9/18) and one *D. novemcinctus* by PCR. Furthermore, 28% of *N. nasua* (5/18) had high parasitemias found by the MHCT test, all which were PCR positive.

We found 50% *N. nasua* (9/18), 56% *S. cay* (5/9) and 40% *C. thous* (2/5) were positive for *Leishmania* spp. All wild mammals tested were negative by hemoculture. By PCR, 22% (4/18) of *N. nasua*, 56% (5/9) of *S. cay* and 20% (1/5) of *C. thous* were positive. Serology testing for *Leishmania* spp. demonstrated that 44% (8/18) of *N. nasua*, and 20% (01/05) of *C. thous* were positive. In *N. nasua* we observed 6% (1/18) that were seronegative but PCR positive. Similarly, we observed discordant results in 28% (5/18) *N. nasua,* which were PCR negative and serology positive for *Leishmania* spp. (Table 2).

Co-infection were observed in 28% (5/18) of *N. nasua* for all three trypanosomatids investigated; in 17% (3/18) of *N. nasua* and 40% (2/5) of *C. thous* we found co-infection with two parasites; and 15% (4/18) of *N. nasua*, 56% (5/09) of *S. cay* and one *D. novemcinctus* were infected by only one trypanosomatid (Table 3). In addition 33% (6/18) of *N. nasua,* 60% (3/5) of *C. thous,* 44% (4/9) of *S. cay,* and all *E. sexcinctus* and *D. azarae* sampled were negative for all parasites investigated (Table 3).

**Table 3:** Co-infection of *Trypanosoma cruzi*, *Trypanosoma evansi* and *Leishmania* spp. from wild mammals sampled in the Urucum settlement, Mato Grosso do Sul state, Brazil in 2015.

Co-infection	Wild Mammals Positives No. (%)					
	*Nasua nasua*	*Sapajus cay*	*Cerdocyon thous*	*Euphractus sexcinctus*	*Dasyprocta azarae*	*Dasypus novemcinctus*
TC	1 (6)					
TC + LEISH	1 (6)		2 (40)			
TC + TE						
TC + TE + LEISH	5 (28)					
TE	2 (11)					1 (100)
TE + LEISH	2 (11)					
LEISH	1 (6)	5 (56)				
Negative	6 (33)	4 (44)	3 (60)	2 (100)	1 (100)	

## 4 Discussion

This study demonstrated that *T. cruzi*, *T. evansi* and *Leishmania* spp. circulate in domestic dogs and wild mammals in Urucum settlement in the extreme west of Brazil. The high occurrence of co-infections in the majority of domestic dogs (73%) is noteworthy. Their role as maintenance hosts and sentinels of these three parasites should be carefully observed within Mato Grosso do Sul border region. Among the wild mammals sampled, *N. nasua* proved to be a key species, where overall 67% were positive with one or more species of parasite and 44% were co-infected. *Cerdocyon thous* was also co-infected by *T. cruzi* and *Leishmania* spp., demonstrating the importance of carnivores in the maintenance of these parasites in sylvatic cycles. Although the most common scenario is to find hosts infected by multiple parasites, the numerous variables involved may be related to different outcomes. Co-infections may alter the frequency or occurrence of infection, as well as the pathogenicity to the hosts, consequently impacting transmission dynamics (Budischak et al., 2012; Enriquez et al., 2016; Fountain-Jones et al., 2017).

Two thirds of dogs at Urucum settlement were positive for *T. cruzi*, half of dogs were positive by PCR with high parasitemias observed in four dogs: one TcI, two TcIII and one TcIII/TcV mix. Primarily, TcI and TcIII have been identified in wild mammals (Brenière et al., 2016; Barros et al., 2017), as observed in the Brazilian Pantanal and in the neighbouring Paraguayan Chaco (Herrera et al., 2008; Acosta et al., 2017). However, TcI and TcIII have also been also reported infecting humans, domestic dogs and synathropic wild mammals in domestic and peridomestic cycles in Bolivia, Paraguay, Colombia, Venezuela and the Amazon (Yeo et al., 2005; Barnabé et al., 2011; Brenière et al., 2016; Enriquez et al., 2016; Barros et al., 2017).

Our data suggests that TcV is more geographic widespread than previously thought. This DTU seems to be associated with human rather than dogs, and wild and synathropic mammals, mainly in Bolivia, Paraguay and Argentina (Barnabé et al., 2011; Monje-Rumi et al., 2015; Brenière et al., 2016). Nevertheless, this DTU has been reported also in dogs of Argentina, Bolivia and northern Brazil (Brenière et al., 2016; Enriquez et al., 2016). In wild mammals, TcV has been identified in six orders in seven countries of South America (Brenière et al., 2016). Importantly, the Gran Chaco region which borders the study location, has been proposed as the origin of TcV, where it is commonly found (Perez et al., 2013).

The different DTUs found infecting dogs in our study may be related to their predation of wild mammals and/or close contact with the *T. cruzi* enzootic environment. In the Gran Chaco, armadillos' species have been reported as the main synathropic sylvatic host of TcIII (Alvarado-Otegui et al., 2012; Acosta et al., 2017). Since armadillos were one of the most cited animals hunted by the dogs, these animals may be getting infected through the oral route. TcV has been found infecting small mammals such as *Monodelphis* sp. and *Rattus rattus* in Bolivia, and TcI was recorded parasitizing *Didelphis albiventris* and *N. nasua* in Gran Chaco and Pantanal region, respectively (Herrera et al., 2008; Alvarado-Otegui et al., 2012). Circulating *T. cruzi* in these wild mammals’ could infect dogs when they hunt or feed on these wild mammals. However, the vector mediated transmission should not be ruled out in enzootic areas, and dogs in the Urucum settlement could be infected by this route, as well as the ingestion of infected triatomine bugs (Montenegro et al., 2002; Reinthinger et al., 2005). Because dogs develop a chronic form of Chagas disease with a low number of circulating parasites (Pineda et al., 1998; Araújo et al., 2002), the source of infection for triatomine vectors is likely low, and therefore the risk to humans unlikely. Co-infections with multiple DTUs in dogs, has been previously found; TcI/TcII was reported in dogs near Serra da Canastra National Park, southeast region of Brazil (Rocha et al., 2013). Dogs have also been documented as co-infected in Colombia by TcI/TcII and TcI/TcIV (Ramírez et al., 2013), which are characteristic genotypes of domestic and sylvatic transmission cycles.

In this study, half of dogs were infected with *Leishmania* spp. The region of Corumbá is an important focus for American Visceral Leishmaniasis in Brazil since the 80's (Nunes et al., 1988; Correa Antonialli et al., 2007). While no dogs or wild mammals were positive by hemoculture, the high prevalence found through PCR and serology indicate that these animals may constitute a source of infection to sand flies. In fact, dogs play a central role in the epidemiology of leishmaniasis while they do not present high parasitemias in the blood, they are main reservoirs due to high parasitic load in the skin (Ciaramella et al., 1997; de Queiroz et al., 2011). The occurrence of *Leishmania* spp. in *N. nasua*, *S. cay* and *C. thous* reinforces the enzootic characteristic of this parasite in the study area. This is the first record of *Leishmania* infecting *N. nasua* and *S. cay* in the central-western region of Brazil, since *C. thous* was previously reported by Mello et al. (1988). Path Analysis supported that consumption of wild mammals was positively correlated with the infection in dogs by *Leishmania* spp. These findings support previous findings regarding oral infection as an important route of transmission of trypanosomatids (Herrera et al., 2011; Roque and Jansen, 2014).

We found a high prevalence of *T. evansi* in dogs (73%) and *N. nasua* (50%), and our results support previous findings by Silva et al. (1995, 1996) and Herrera et al. (2004), where a high occurrence of infected dogs and *N. nasua* were found in the nearby Pantanal region. The high prevalence of *T. evansi* in the study area suggest that *N. nasua* is an important reservoir host for this parasite, as seen in the other sites in the Pantanal wetland (Nunes et al., 1993; Herrera et al., 2004). This high prevalence in *N. nasua* could affect the infection in dogs. In enzootic areas, dogs that live in close contact with wild reservoirs are often infected with *T. evansi* (Franke et al., 1994; Herrera et al., 2004).

In dogs, the main clinical signs of *T. evansi* infection is severe anemia, which was seen in our study by the association between *T. evansi* infection and mucous pallor. Nevertheless, as reported by other authors, intermittent fever, subcutaneous edema, blindness, lethargy, haemostatic changes, and a lack of coordination may also be observed in dogs infected by *T. evansi* (Singh et al., 1993; Brandão et al., 2002).

Although we did not find a significant association among co-infections in dogs, our data showed that the co-infection by *Leishmania* spp. and *T. cruzi* were related to onychogryphosis. Additionally, we found that infection with *Leishmania* spp., *T. cruzi* and *T. evansi* were related to skin lesions. A natural case of co-infection by the hemoparasites *Leishmania chagasi* and *T. evansi* in one dog was reported in the south western of Mato Grosso do Sul (Savani et al., 2005). However, to the best of our knowledge, this study is the first to registered co-infection by *T. cruzi*, *T. evansi* and *Leishmania* spp. in dogs. When managing the conservation of wild mammals, it is important to consider that co-infection by *T. cruzi* and *T. evansi* influence the health of free-living *N. nasua* (Alves et al., 2011; Olifiers et al., 2015).

We found discordant results between serological and molecular assays indicating differences in infection. The chronicity of *T. cruzi* and *Leishmania* spp. infection is exemplified in our study by several dogs and *N. nasua* that were serology positive and PCR negative. In the case of *T. evansi*, the low occurrence of cryptic infection in dogs is probably associated with fatal characteristic of this disease.

The occurrence of seronegative, but PCR positive dogs and *N. nasua* could be associated with the initial acute infection, characterized by the low level of serum immunoglobulins. We observed that half of the dogs and 17% of *N. nasua* displayed *T. cruzi* in the blood. *Trypanosoma cruzi* parasitemia is higher in the acute phase than in the chronic phase (Pineda et al., 1998; Araújo et al., 2002). Alternatively, the presence of *T. cruzi* in the blood could also be due to reinfection by the same or different DTUs (Machado et al., 2001). This might also be seen in *T. evansi* and *Leishmania* spp. infected animals. The high occurrence of PCR positive dogs and *N. nasua* suggests active transmission in the studied area.

## 5 Conclusions

In conclusion, our results reveal the importance of monitoring trypanosomatids in dogs in cross-border Brazil-Bolivia due to their susceptibility. We found that dogs in the Urucum settlement serve as incidental hosts in the active sylvatic cycle of *T. cruzi*, *T. evansi* and *Leishmania* spp. which was seen by the infected wild mammals. The high prevalence of these three parasites found in dogs demonstrates that they are important domestic reservoirs and potentially important sentinels to human infection in the studied area. Finally, Urucum settlement could be considered a *hotspot* to emergent neglected diseases caused by trypanosomatids due to the high occurrence of the three trypanosomatids investigated.

## Ethical approval

All field procedures and laboratory studies were conducted in accordance with a license granted by the Biodiversity Information and Authorization System of the Chico Mendes Institute for Biodiversity Conservation (license number 47821-3). The present study was approved by the Ethics Committee for Animal Use of Dom Bosco Catholic University, Campo Grande, MS (license number 011/2015). All dog owners signed a consent form before dogs were sampled.

## Declarations of interest

None.

## Conflict of interest

The authors have declared that no competing interests exist.
